# Shape completion in the dark: completing vertebrae morphology from 3D ultrasound

**DOI:** 10.1007/s11548-024-03126-x

**Published:** 2024-05-15

**Authors:** Miruna-Alexandra Gafencu, Yordanka Velikova, Mahdi Saleh, Tamas Ungi, Nassir Navab, Thomas Wendler, Mohammad Farid Azampour

**Affiliations:** 1 Chair for Computer Aided Medical Procedures & Augmented Reality, Munich, Germany; 2 School of Computing, Queen’s University, Kingston, ON Canada; 3 Clinical Computational Medical Imaging Research, Department of Diagnostic and Interventional Radiology and Neuroradiology, University Hospital Augsburg, Augsburg, Germany; 4 Munich Data Science Institute, Munich, Germany; 5 Munich Center for Machine Learning, Munich, Germany

**Keywords:** Ultrasound imaging, 3D shape completion, Physics-based data generation, Visualization enhancement

## Abstract

****Purpose**:**

Ultrasound (US) imaging, while advantageous for its radiation-free nature, is challenging to interpret due to only partially visible organs and a lack of complete 3D information. While performing US-based diagnosis or investigation, medical professionals therefore create a mental map of the 3D anatomy. In this work, we aim to replicate this process and enhance the visual representation of anatomical structures.

****Methods**:**

We introduce a point cloud-based probabilistic deep learning (DL) method to complete occluded anatomical structures through 3D shape completion and choose US-based spine examinations as our application. To enable training, we generate synthetic 3D representations of partially occluded spinal views by mimicking US physics and accounting for inherent artifacts.

****Results**:**

The proposed model performs consistently on synthetic and patient data, with mean and median differences of 2.02 and 0.03 in Chamfer Distance (CD), respectively. Our ablation study demonstrates the importance of US physics-based data generation, reflected in the large mean and median difference of 11.8 CD and 9.55 CD, respectively. Additionally, we demonstrate that anatomical landmarks, such as the spinous process (with reconstruction CD of 4.73) and the facet joints (mean distance to ground truth (GT) of 4.96 mm), are preserved in the 3D completion.

****Conclusion**:**

Our work establishes the feasibility of 3D shape completion for lumbar vertebrae, ensuring the preservation of level-wise characteristics and successful generalization from synthetic to real data. The incorporation of US physics contributes to more accurate patient data completions. Notably, our method preserves essential anatomical landmarks and reconstructs crucial injections sites at their correct locations.

**Supplementary Information:**

The online version contains supplementary material available at 10.1007/s11548-024-03126-x.

## Introduction

US imaging provides a noninvasive, radiation-free, and low-cost way to observe internal structures and organs in real time. While valuable, this modality has its own limitations such as reduced field of view, user dependence, and the presence of artifacts.

Due to the underlying physical properties of US imaging, highly reflective structures such as bones introduce shadows occluding tissue below them. In contrast, imaging techniques like CT and MRI provide comprehensive representations of anatomical structures without angle dependence and significantly fewer occlusion artifacts. Consequently, interpreting US images can be notably more challenging [1].

When using US in a conventional fashion to extract anatomical information needed for diagnosis or intervention, medical professionals must rely on their expertise to mentally reconstruct the 3D shape of the organ or structure from partial US views. This not only adds to the time and effort of the diagnostic process but also presents a learning challenge for young professionals. Our objective is to assist in this process by enhancing the ultrasound view with the complete 3D shape and facilitating a rapid and more intuitive understanding of the anatomy. We employ 3D shape completion techniques [2] to deduce the complete contour of organs based on the partially visible anatomy in an US sweep, ensuring that salient structural details are preserved. In this manner, not only do we assist professionals, but we also translate this intricate cognitive task into a format machines can process.

Various deep learning (DL) techniques have been proposed for 3D shape completion. These methods use a combination of local and global features with diverse representations. The early methods, like Point Completion Network (PCN) [3] and TopNet [4], employ folding operations, offering a rough reconstruction of the shape modeled as a point cloud (PC). Later, DeepSDF [5] proposed to leverage continuous signed distance fields to learn about shape categories and improve quality. PoinTr [6] then introduced a technique to predict only the missing region and concatenate the inputs and outputs of the model to produce the final completion. In one of the newer methods, Variational Relational Completion Network (VRCNet) [7], a probabilistic approach is adopted. Here, a shape prior distribution is learned across various object classes. Following this learning, the shape completion is derived using Maximum a Posteriori (MAP) estimation, where the input partial PC serves as the observed data.

This state-of-the-art shows that training DL methods for shape completion requires substantial datasets to achieve optimal results. In the computer vision realm, where all the cited work originates, CAD models of objects are often employed to produce realistic occlusions, thereby creating extensive training datasets. Extending this paradigm to the medical domain, our objective is to generate CAD-inspired representations of partial views in US by simulating physics-based occlusions. Generating synthetic data in this manner holds particular promise in medical areas where US is limited in clinical settings, where patient data is scarce or hard to get.

One such area is the examination and intervention on the spine, which is extensively explored in research but has yet to be fully established in clinical practice using US [8] despite its high potential to reduce radiation exposure to patients and medical personnel. The challenge with spine US scans lies in their limited visibility; only the posterior surface of the spine can be imaged. These scans are primarily affected by acoustic shadowing, preventing the US beam from reaching deeper vertebral structures below the spine surface. This limitation complicates the operator’s comprehension of the entire spine anatomy.

Shape completion of the partially visible vertebra can help in overcoming this limitation. Inspired by VRCNet, we propose to use a point cloud-based probabilistic method that takes advantage of preexisting 3D imaging, such as computer tomography (CT), which offers comprehensive 3D shape details, to understand shape priors. The proposed method learns fine 3D PC geometries of vertebrae and predicts consistent and detailed PCs for the occluded regions.

For the training of our model, we introduce a unique, fully automated pipeline for generating synthetic data. We generate physics-based synthetic data that mimics US characteristics, bridging and facilitating the application of multiple shape completion techniques in medical contexts, otherwise unfeasible due to lack of access to paired US/CT data. When integrated with the proposed 3D PC reconstruction network, our pipeline enables the completion of vertebrae shapes from 3D US data. Through shape completion we introduce a new perspective to tackle US data interpretability, and to the best of our knowledge propose a first work in the direction of 3D anatomical shape completion from ultrasound scans. In summary, the contributions are threefold:

1. We develop a synthetic data generation pipeline that produces realistic, US-consistent partial views of lumbar vertebrae.
2. We introduce a 3D shape completion pipeline for lumbar vertebrae.
3. We evaluate our method’s shape completion capabilities on synthetic and CT-US patient data, and report standard computer vision metrics as well as anatomy-based ones.

## Materials and methods

### Synthetic data generation

In a common computer vision pipeline, training data for shape completion is created by generating realistic occlusions of objects using CAD models, e.g., by ray-casting from different camera positions around the object. Much like this approach, our synthetic data generation pipeline utilizes high-resolution abdominal CT scans with vertebral masks to generate partial PCs resembling vertebrae visibility in US.

**Fig. 1:**
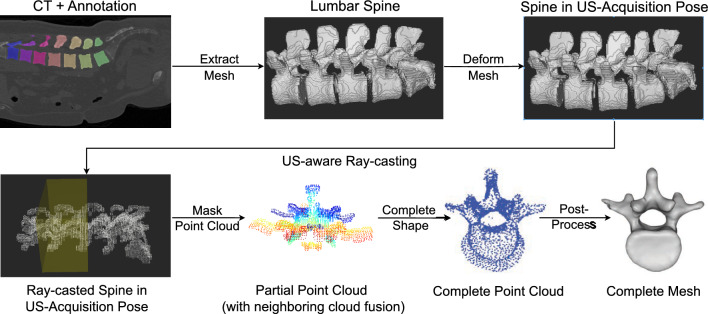
Overview of the training pipeline of our proposed method. First data generation is performed, followed by shape completion and post-processing

Three main milestones need to be achieved to generate a large amount of realistic, US-consistent partial views of the vertebrae only using an abdominal CT scan. First, we need to account for the multitude of possible patient positioning during the US acquisition. Second, we need to generate partial views of the spine that adhere to spine US acquisition techniques and their field of view and faithfully replicate the effects of US-characteristic artifacts such as acoustic shadowing or scattering. Lastly, our method must account for potential inaccuracies stemming from the error-prone, challenging task of vertebrae classification and annotation in US. Figure 1 displays the complete data generation pipeline described in detail in the following. For an algorithmic overview, please refer to the online supplementary material.

#### Accounting for multiple spine curvatures

Patient positions during ultrasound screening vary depending on the target anatomy and spine region. While sitting is typical for visualizing the interlaminar space, the prone position aids lumbar facet joint access. To encompass this range of spine curvatures, we enhance the spine meshes from CT to produce varied realistic curvatures for training. This provides the network with diverse vertebrae poses during training, increasing the robustness of the shape completion model. When adjusting the spine’s curvature, it is vital to consider the spine’s physical constraints. A step-by-step algorithm of how we achieve multiple spine curvatures through realistic spine model deformations can be found in the online supplementary material. This algorithm follows the approach proposed by Azampour et al. [1]

#### Generation of US-consistent partial views of the spine

**Fig. 2:**
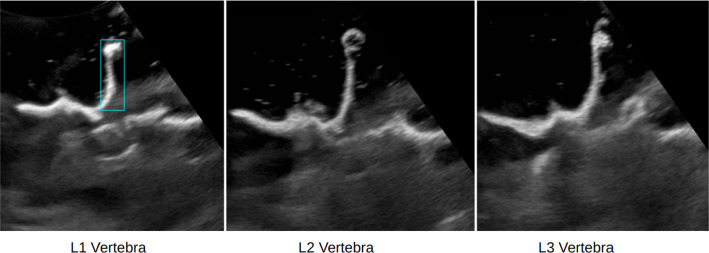
US scan of L1, L2, and L3 vertebrae levels of a spine phantom. These images exemplify the partial view of the vertebral arch as well as US-specific artifacts. We can see the effects of acoustic shadowing in the partially visible spinous process, highlighted with a bounding box on the left image

Spine US scans, whether transverse or paramedian, typically display only the vertebral arch’s surface. Figure 2 showcases partial vertebrae in US, displaying vertebrae L1, L2, and L3 of a lumbar phantom. Structures like the spinous process, the laminae, the articular processes, and the transverse processes are only partially visible. Parts of these structures are rendered invisible due to a large angle of incidence between the direction of the US wave and the respective tissue. In other cases, they are occluded by the surrounding structures due to the effect of acoustic shadowing. Notably, the vertebral body is frequently fully shadowed.

Beyond acoustic shadowing, US exhibits scattering, causing minor displacements in the way some tissue appear on the image. This artifact amplifies noise and occlusions in an US vertebral view. In what follows, we will showcase how our technique produces partial spine views consistent with these US-specific characteristics.

***Angle of incidence-aware ray-casting***

US visibility hinges on the interaction and reflection of ultrasound waves with internal body structures. A pivotal factor is the angle of incidence—the angle at which the US wave hits the tissue. When this angle is below 90^∘^, the US beam reflects, capturing and displaying the signal. Yet, at angles over 90^∘^, especially when tissue boundaries align with the beam, the signal goes undetected, omitting the tissue interface from the display. For authentic, US-consistent PCs, accounting for this phenomenon is vital.

We simulate the transversal US acquisition on spine meshes to produce partial views. Addressing the angle of incidence, we employ a technique that is cognizant of it. We strategically position the virtual rendering camera over each spinous process, casting rays to identify visible points. In this process, we compute the angle between each ray and the tissue plane and omit points with incidence angles of $\ge 90^{\circ }$. The impact of this technique is more significant degrees of occlusion, thereby enhancing the resemblance of the resulting PC to a US view.

***Account for ultrasound scattering***

To emulate US scattering—an effect where US beams register off-plane echoes, we simulate off-plane signals by subtly shifting the spine perpendicularly to the incident ray direction and ray-casting it alongside the originally positioned spine. From this mesh, we then retain points unobstructed by the shift. The resultant PC exhibits more shadows, thus mirroring an ultrasound view of the spine.

#### Masking spine into separate vertebrae views

Our data generation pipeline concludes with dividing the spine into individual vertebra views, resulting in five vertebral PCs serving as partial network inputs. Segmenting spinal ultrasound into distinct vertebrae levels is challenging and prone to errors. To the best of our knowledge, no method can accurately differentiate between vertebrae levels in ultrasound images. Hence, our approach aims for realistic completions without relying on this specific information. To increase our method’s robustness, we augment our data by performing neighboring cloud fusion. This process merges the PCs from one vertebra with points from directly adjacent vertebrae.

### Vertebrae shape completion

For the completion of vertebrae shapes, we employ a probabilistic approach based on VRCNet [7].

The shape completion pipeline consists of two networks that follow the variational autoencoder architecture. They are trained end to end using a composite loss function that incorporates two distinct components: the Kullback–Leibler (KL) divergence loss and the Chamfer Loss (CL) as reconstruction loss. The two networks are (1) Probabilistic Modeling Network (PMNet) and (2) Relational Enhancement Network (RENet) (for details see online supplementary material).

PMNet employs probabilistic modeling to yield initial coarse completions by decoding global features. During training, it grasps the prior distribution of vertebrae shapes, capturing essential details about shape, size, and symmetry. At inference, the model refines the shape using observed data and the posterior distribution, allowing for patient-tailored completions that respect both general anatomical priors and unique characteristics of the individual’s spine.

RENet operates on both the partial and coarse-complete PCs produced by PMNet. Using an encoder–decoder design enhanced with self-attention modules, it can aggregate point features across various scales. This is crucial for detailed vertebral completions, preserving input nuances from the partial cloud while recovering specific occluded anatomical features of individual anatomies.

The proposed method generates the completed shape as a PC. To better visualize the results, we apply a step of post-processing and generate a vertebral mesh based on Poisson Surface Reconstruction.

### Datasets description

**Large-scale vertebrae segmentation challenge dataset 2020** [9–11] One dataset utilized in our study, referred to as the VerSe20, comprises abdominal CT scans that contain detailed annotations and classifications of vertebrae. Specifically, VerSe20 includes 125 lumbar vertebrae, evenly distributed across different levels, with 25 vertebrae per level. For our work, VerSe20 serves as the foundational dataset of our synthetic data generation pipeline.

**Paired US/CT patient data** The patient data comprises a total of two paired US/CT scans [12]. The ultrasound sweeps were obtained while the patient was in a sitting position, which is the standard pose for epidural injections. Through this data we assess the applicability of our method for this procedure. To input this data into the shape completion network, we first perform a manual annotation of the bone in ultrasound, followed by a rough separation of the vertebrae. To generate the ground truth (GT) complete vertebral shape, we apply the automatic spine segmentation method proposed by Payer et al. [13], and obtain vertebra-wise segmentations.

**Phantom dataset** To evaluate the shape and pose preservation of landmarks visible in the initial US, we use a lumbar spine phantom. This phantom consists of all five lumbar vertebrae as well as the intervertebral disks and the sacrum.

### Shape completion metrics

**General metrics** In our evaluation process, we utilized three key metrics: CD, Earth Mover’s Distance (EMD), and F1-score (F1). The CD, widely employed in the computer vision community, calculates the point-to-point distance between two PCs: one representing the completed shape and the other the ground truth shape. To enhance interpretability, we scaled our CD values by a factor of $10^{4}$ following the approach of VRCNet. EMD measures the dissimilarity between two shapes by quantifying the minimum amount of work required to transform one shape into the other. Lastly, to address the impact of outliers, we incorporated an adapted version of the F1-score, as proposed by Knapitsch et al. [14]. This metric represents the harmonic mean of precision and recall, and serves as an additional measure of our methodology’s performance.

**Anatomy-specific metrics** Moving away from the general metrics, we introduce two anatomy-specific metrics. The spinous process is typically visible in ultrasound scans, making it a key reference point for our shape completion network. We aim to maintain its integrity and make sure it is placed at the appropriate location. To assess this, we calculate the Spinous Process Chamfer Distance (SP-CD) metric. This metric involves comparing two point sets generated by manually annotating the centerline of the spinous process surface in both the input and the completion. This measurement allows us to evaluate the fidelity of the spinous process preservation and placement in the completed shape.

Another anatomical landmark is the facet joint, which connects neighboring vertebrae. To ensure that the facet joints are preserved in the 3D completion at the correct position, we measure the distance between the facet joint’s center in the reconstruction and its correct location from the CT-based ground truth.

## Experiments

Our study begins with an evaluation of our proposed method and comparison of two shape completion approaches: the network described in 2.2 and the approach proposed by the PCN work [3]. This exemplifies the capability of our pipeline to integrate any, and therefore the most suitable point cloud-based shape completion approach for the task at hand. Next, we conduct an experiment dedicated to verifying how well the visible anatomical landmarks in US are preserved in the completion. Lastly, we conduct two ablation studies, which aim to investigate the impact of the two US-related steps in the data generation pipeline, i.e., the US physics and the neighboring cloud fusion, on the accuracy of our results. To evaluate the suitability of each model for shape completion in patient ultrasound images, we also evaluate using the paired US/CT patient dataset, the details of which are outlined in Sect. 2.3. Our analysis includes both quantitative and qualitative results for a comprehensive understanding of the outcomes.

### Experimental setup of our method

We split the VerSe20 dataset subjects based into 60%–20%–20% for training, validation, and testing. For each experiment, we train for 100 epochs. The optimization uses the Adam optimizer with a learning rate set at 0.0001. For training, a batch size of 8 is utilized, whereas during testing, a batch size of 2 is employed. The training procedures are executed on an NVIDIA GeForce RTX 2080 GPU. The training durations for the proposed methodology and the two ablation studies are roughly 15 h, 5 h, 15 h, respectively. During the inference stage, the shape completion process for a batch comprising two vertebrae, on average, takes 0.22 s.

### Anatomical landmarks preservation

In evaluating our method, we place special emphasis on the vertebral arch, given its partial visibility in US. We want to ensure that the shape and pose of the anatomical landmarks in the vertebral arch such as the spinous process and the lateral process are preserved. To evaluate if these landmarks are preserved, we compute the anatomy-specific metrics (Sect. 2.4) on our phantom dataset. We choose the phantom instead of the patient data for this experiment, since it facilitates the correct identification of the landmarks’ pose.

### Ablation study

In our ablation study, we systematically explore the impact of individual steps in synthetic data generation on accurate patient shape completion.

**Synthetic data without considering US physics** This experiment assesses the significance of incorporating US physics [15] into synthetic data generation. For this experiment, we do not consider US-specific acquisition modalities nor US-artifacts while generating the data. This translates to a simplified ray-casting process, omitting considerations of angle of incidence and completely bypassing the scattering step.

**Synthetic data without performing neighboring cloud fusion** This experiment explores the network’s performance on inaccurately separated vertebrae from patient data when trained solely on point clouds on which neighboring cloud fusion augmentation has not been performed. These point clouds therefore contain only points relevant to the specific vertebra without including points from neighboring structures. To achieve this, we omit the masking step in the data generation pipeline and, based on the CT annotations, generate vertebrae PCs that are meticulously separated from neighboring vertebrae.

## Results

### Evaluation of proposed methodology on synthetic and patient data

**Fig. 3:**
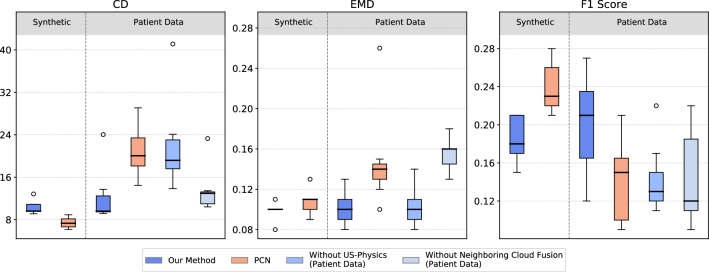
Performance comparison (in terms of Chamfer Distance (CD), Earth’s Mover’s Distance (EMD), and F1-score) of our full pipeline with two different shape completion approaches (VRCNet (blue) and PCN (orange))on synthetic and patient data, as well as results of the ablation studies

The plots in Fig. 3 compare the performance of the model trained on synthetic data both on the generated test set and patient data. Generally, the results on patient data show a larger variance and slightly lower accuracy. However, the differences of our method in all three metrics are relatively small, suggesting that our network can generalize from synthetic to patient data.

Additionally, we compare to PCN[3], which, trained in the same manner, achieves comparable or even increased accuracy in the case of synthetic data. This demonstrates the interchangeability of the shape completion approach in our pipeline. However, unlike the proposed shape completion network, the PCN is not able to generalize well to the patient data. This, by comparison, demonstrates the suitability of our chosen shape completion method for clinical applications.

### Preservation of landmark pose

**Spinous process** Table 1 displays the dissimilarity between spinous process centerlines in the completion and in the input for each vertebral level. The accuracy only for this landmark is higher than the one for the complete shape. These results demonstrate the ability of the network to preserve the US-visible landmarks in the completion and reconstruct them at the correct position.

**Facet joints** The facet joint reconstruction accuracies measured as the distances between the center of the facet joint in the completion and GT are displayed in Table 1. According to [16], an accuracy error of 5 mm is still acceptable for a successful anesthetic effect for facet joint injections. From our results, three out of four completed facet joint pairs would enable accurate injection delivery, while one pair (between L3 and L4) exceeds this threshold by at most 1.66mm.

**Table 1:** Facet joint reconstruction accuracy measured as the distance between the center of the facet joint in the completion and in the ground truth

Vertebra Level	SP-CD	Left facet joints dist (mm)	Right facet joint dist (mm)
L1	6.81	4.50	5.19
L2	2.00	2.64	4.87
L3	2.88	4.97	3.46
L4	6.09	6.45	7.66
L5	5.88	–	–

### Ablation studies

**Fig. 4:**
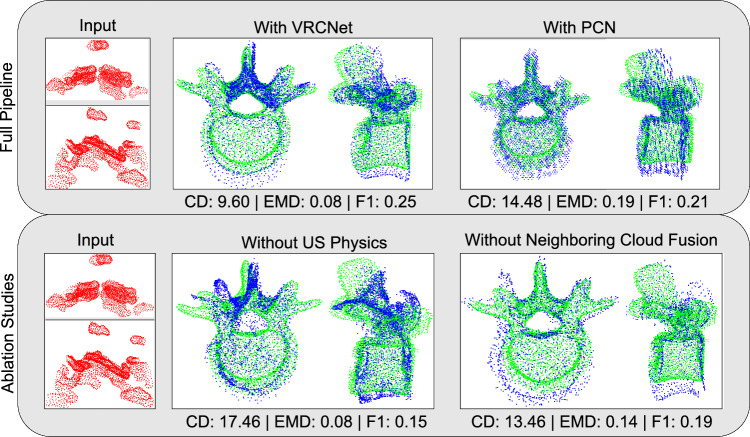
Patient data results obtained with the full pipeline comparing two shape completion networks as well as two ablation studies. Given our partial PC input (red), we compare the reconstruction (blue) with the ground truth (green) and report three metrics: CD, EMD, and F1. We visualized the input and each completed shape PC from two views along the frontal and longitudinal axes

Figure 3 reports the quantitative results of the ablation studies on the patient dataset. Parallely, Fig. 4 shows examples of the qualitative results of our completions. Extensive qualitative results can be found in the online supplementary material.

**Results without US physics** Considering the physics of US during the generation of synthetic data improves the accuracy of shape completion on patient data. As measured by the CD, the accuracy of all completions increases. Specifically, we observe a median difference of 6.79 in the CD metric, indicating a noticeable improvement. This difference is also reflected in the other two scores, however, with a smaller median difference.

Qualitatively, omitting US physics simulation during training data generation leads to completion with unwanted points in the vertebral spinal canal, the area that houses the spinal cord. Additionally, important landmarks such as transverse processes and facets are missing in certain completions, for example, the transverse processes in Fig. 4. Furthermore, the completed shapes of the ablated model resemble less the GT shape, which can be particularly observed when looking at the vertebral body.

**Results without neighboring cloud fusion** The proposed method outperforms the ablation model, an aspect which is reflected in all three metrics. The largest median difference of 0.06 is observed for the EMD score.

In terms of qualitative assessments, the completions of the ablation model are relatively sparse. This is reflected in the low F1 values of this experiment. Moreover, the resulting completions contain multiple points in the spinal canal.

## Discussion and conclusion

In this work, we present a novel technique that addresses the challenge of completing anatomical structures given partial visibility in 3D US. Our method leverages synthetic data that considers US physics and artifacts, ensuring consistency with the partial display of anatomy in US. Moreover, generating this data considers process-specific augmentations such as curvature deformations and neighboring cloud fusion. We specifically apply our shape completion approach to the realm of US-based spine investigation. In this context, the proposed method completes the shapes of vertebrae. We demonstrate the generalizability of the proposed method to patient data, although trained only on synthetic data. This successful generalization emphasizes that our data generation process is realistic and US-consistent.

First of its kind, our proposed approach is capable of completing the shape of the vertebrae without prior patient-specific information, given only the US scan. This is particularly relevant in situations where a diagnostic CT scan is either unavailable or acquiring one is restricted due to factors like radiation concerns, for example, in the case of epidural injections.

The obtained results show promising outcomes, indicating the potential for further exploration in this area. Notably, enhancements in accuracy could be achieved by incorporating additional parameters such as vertebral level or patient BMI. These details could offer valuable contextual cues for the method, aiding in a more precise estimation of the vertebral shape. To advance toward highly accurate, patient-specific outcomes, the introduced approach could be refined during the testing phase by including the patient’s CT scan data. This would provide precise information about the vertebra shapes, improving the performance of the US-based completion.

In clinical settings, highly accurate, patient-specific completions would enable integrating the method into the workflow of spine injection surgeries. An ultrasound-based navigation system that displays the complete vertebral anatomy can assist surgeons in needle placement. For instance, it could help identify the level of the currently visualized vertebra in ultrasound, a very challenging and error-prone task. However, the final injection site confirmation would still rely on the original ultrasound. To optimize this guidance system, it is important to explore suitable rendering techniques and devise an adequate real-time component for use in the operating room.

One current limitation of our work is the fact that it focuses on a single anatomy for shape completion. The ultrasound scan, which includes information about surrounding tissues, organs, and structures, is not fully utilized in the completion process. Incorporating this information could enhance the accuracy, providing cues about the size, pose, or even abnormalities of the vertebral bodies, a structure not often captured in an ultrasound scan. This concept could be gradually extended to other regions of the spine, then to all types of rigid anatomies. Subsequently, devising appropriate methods to model and handle even deformable anatomies would be a relevant research direction.

The proposed method relies on certain reference structures, such as the spinous process, to be correctly segmented in US. This makes our method prone to errors if these structures are absent in the input PC or wrongly segmented. Furthermore, the scope of our study was limited by the size of our dataset. While our research successfully demonstrated a proof of concept, a more comprehensive evaluation of the proposed method’s capabilities necessitates a larger dataset, in particular paired US/CT patient data. A broader, large-scale study would provide a more thorough understanding of the method’s performance across diverse scenarios, such as pathologies, different US acquisition protocols or quality, and further validate its effectiveness.

In conclusion, the proposed method improves the interpretation of US images by enhancing the visualization of anatomical structures in US scans. Mimicking how clinicians envision 3D anatomy, it incorporates prior knowledge of the shape of the target structures, and considers the physics of US imaging. In clinical practice, this technology could facilitate experts to rapidly and intuitively gain better understanding of the anatomy without the need for additional imaging modalities. As an exemplary application, our method completes occluded vertebrae in US spine scans. We show that using synthetic 3D spinal views that consider the nature and artifacts of US imaging for training yields a model that provides consistent results on synthetic and clinical data. Notably, our approach maintains crucial anatomical landmarks in 3D completion, like the spinous process and the facet joints. Overall, this work shows a high potential for detailed lumbar vertebrae visualization and, ultimately, a path to explore toward the replacement of X-ray imaging for spine diagnosis and intervention.

**Supplementary information** An accompanying PDF file containing additional figures, comprehensive result visualizations, and detailed algorithm descriptions is available online alongside our main article.

### Supplementary Information

Below is the link to the electronic supplementary material.

Supplementary file 1 (pdf 5078 KB)

Supplementary file 2 (pdf 2754 KB)

## Data Availability

Available at https://github.com/miruna20/Shape-Completion-in-the-Dark.
