# Supplementary material for: Shape completion in the dark: completing vertebrae morphology from 3D ultrasound
Source: Int J Comput Assist Radiol Surg. 2024 May 15;19(7):1339–47. doi: 10.1007/s11548-024-03126-x (PMC11231015; doi:10.1007/s11548-024-03126-x)
Supplement: Supplementary file 1 — (pdf 5078 KB) [file 11548_2024_3126_MOESM1_ESM.pdf]

Supplementary Material to: Shape Completion in the Dark: Completing Vertebrae Morphology from 3D Ultrasound

Miruna-Alexandra Gafencu[1,4*], Yordanka Velikova[1,5], Mahdi Saleh[1], Tamas Ungi[2], Nassir Navab[1,4,5], Thomas Wendler[3,1], Mohammad Farid Azampour[1,5]

[1*]Computer-Aided Medical Procedure and Augmented Reality (CAMP), CIT, Technical University of Munich,Garching bei Muenchen, Germany.
[2]School of Computing, Queen's University, Kingston, Ontario, Canada.
[3]Clinical Computational Medical Imaging Research, Department of Diagnostic and Interventional Radiology and Neuroradiology, University Hospital Augsburg, Augsburg, Germany.
[4]Munich Data Science Institute, Munich Germany.
[5]Munich Center for Machine Learning, Munich, Germany.

*Corresponding author(s). E-mail(s): miruna.gafencu@tum.de;

This supplementary material includes further details on implementation, datasets and a comprehensive overview of our qualitative results.

1 Implementation Details

1.1 Synthetic data generation pipeline

1.1.1 Angle of incidence-aware Raycasting

To better understand the visual impact of the data generation pipeline on the resulting vertebral point cloud, we display a comparison of the spine mesh ray-casting with and without considering the angle of incidence in Figure 1. We observe that considering the angle of incidence leads to point cloud with more shadows, reflecting the shadowing effects in US.

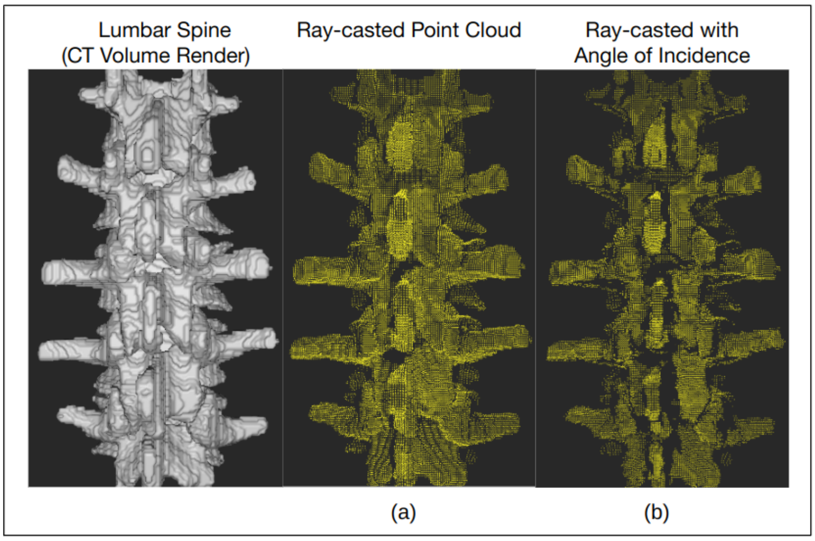

Fig. 1 Comparison of spine mesh ray-casting when (a) the angle of incidence is not considered (b) the angle of incidence is considered. The resulting point cloud contains more shadows and is, therefore, more similar to the US view of the spine.

1.1.2 Account for Ultrasound Scattering

To simulate the effect of ultrasound scattering, we have empirically defined shift values symmetrically along the lateral axis and asymmetrically on the posterior-anterior axis, detailed in Table 1. The combined mesh of the centered (blue) and shifted spine (orange) is presented on the left side of Figure 2. From this mesh, we retain points of the centered mesh unobstructed by the shift. The resultant point cloud, exhibiting more shadows, mirrors an ultrasound spine image, as visualized on Figure 2's right side. The occlusion extent and areas are shift-dependent. To diversify our synthetic output, we utilize all shift pairs from Table 1, yielding nine unique point clouds per dataset.

	**Shift 1**	**Shift 2**	**Shift 3**
**Lateral axis (symmetrical)**	±5mm	±7mm	±10mm
**Anterior-posterior axis (asymmetrical)**	-1mm	-5mm	-10mm

Table 1 Shift values used to account for the effect of scattering in US. We use all possible pairs from these values, a total of 9 shifts per mesh for the data generation.

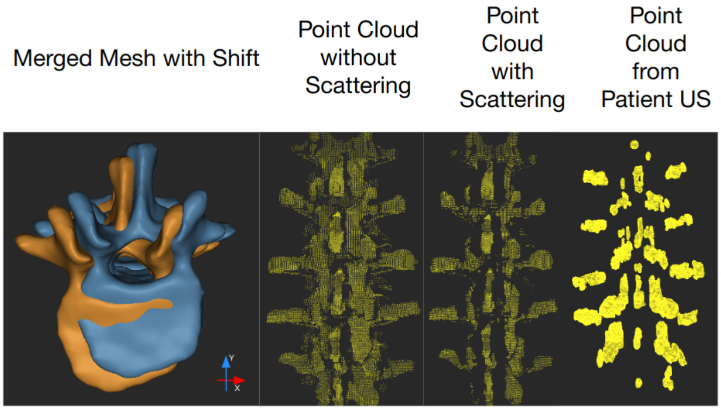

Fig. 2 Left: Example of merge between centered in blue and shifted mesh in orange by -7mm along the lateral axis (x-axis) and -5mm along the anterior-posterior axis (y-axis). Right: Comparison of ray-casted point cloud with and without scattering. We observe that the scattered version displays more shadowed areas.

1.1.3 Masking spine into separate vertebrae views

To perform the neighboring cloud fusion augmentation, we sequentially place a bounding box centered on each vertebra's center of mass as visualized in Figure 3. We then extract all points within this bounding box to create the input point cloud. As exemplified in Figure 3 where the box is centered on vertebra level L3, this technique allows us to collect and merge a small number of points from neighboring vertebrae (here L2 and L4) with the points from the current vertebra.

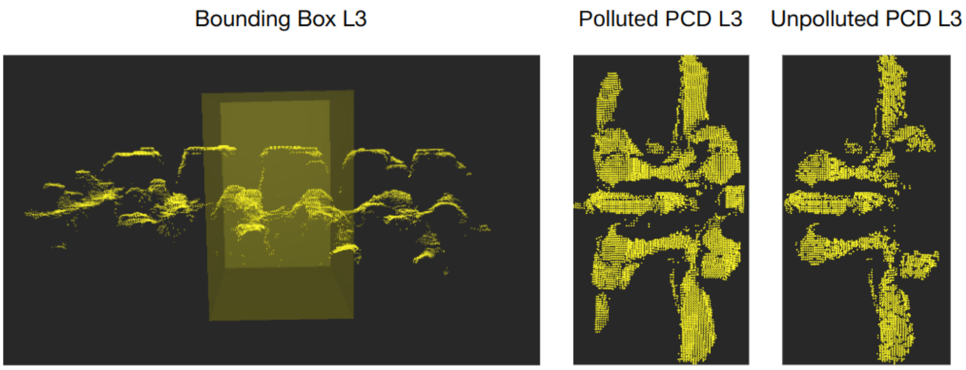

Fig. 3 The spine point cloud is masked to obtain vertebra-wise point clouds. On the left, the masking process is visualized, in which we sequentially place a bounding box centered on each vertebra and select all points within. The middle and right image compare the results of this process which we call neighboring cloud fusion with the results of omitting it.

1.2 Vertebrate Shape Completion Network Architecture

The architecture of the shape completion method consists of two networks. (1) Probabilistic modeling network (PMNet) displayed in Figure4 and (2) Relational Enhancement Network (RENet) shown in Figure5.

(1) The PMNet consists of two paths. The first path, known as the reconstruction path, takes a complete shape as input and reconstructs it. Simultaneously, the completion path generates a coarse point cloud from the incomplete input. These pathways are designed in an autoencoder fashion. They share weights, and the reconstruction distribution is used to regularize the completion one. (2) The RENet is designed as an encoder-decoder structure with self-attention building blocks.

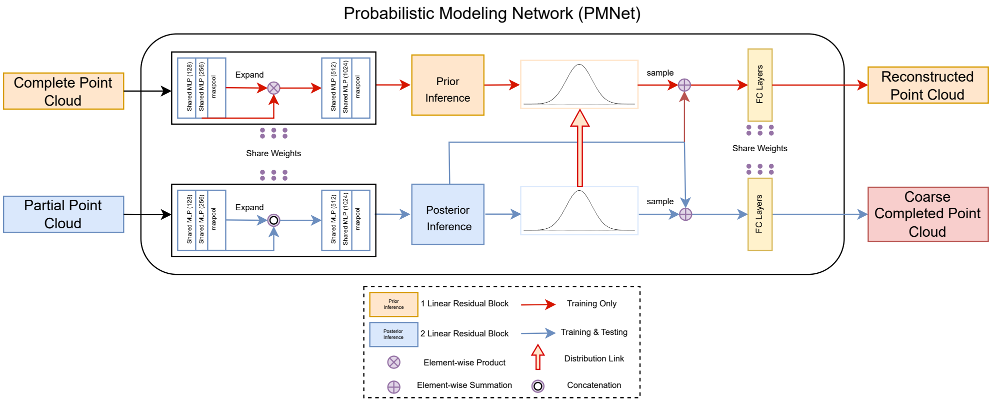

Fig. 4 Overview of Variational Relational Completion Network (VRCNet)'s Probabilistic Modeling Network (PMNet), which generates the coarse complete point cloud at inference time based on the reconstruction latent distribution learned at training time. This figure was adapted from the original paper [1].

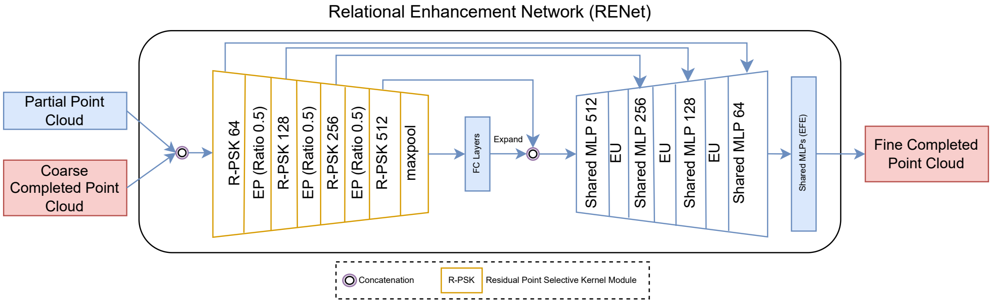

Fig. 5 Overview of VRCNet's Relational Enhacement Network (RENet), network that generates the fine completion by using self-attention building blocks to recover local details. This figure was adapted from the original paper [1].

1.3 Baseline Network as Point Completion Network

We consider the PCN[2] as the baseline architecture for shape completion and compare our method to it. We train the network with the chamfer distance loss for 400 epochs with a learning rate of 0.0001.

2 Dataset details

2.1 Phantom Data

The lumbar spine phantom utilized in this work is shown in Figure 6. It contains the five lumbar vertebrae as well as the sacrum and intervertebral disks. We acquired a 3D US scan with a transverse probe orientation by using ACUSON Juniper (Siemens Healthineers, Erlangen, Germany) with a 5C1 convex probe. This probe is mounted on a 7-axis robot of the model KUKA LBR iiwa 7 R800 manipulator (KUKA Roboter GmbH, Augsburg, Germany).

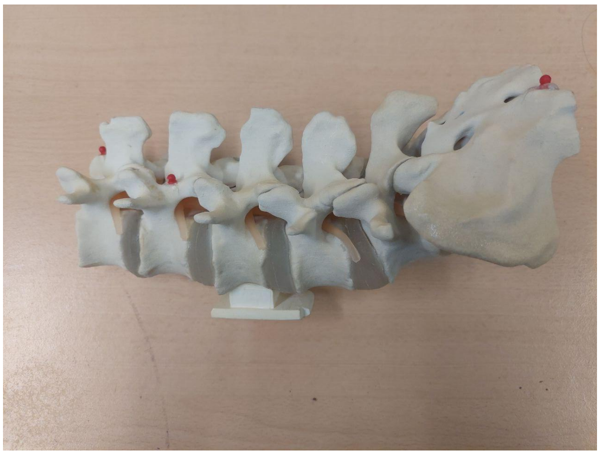

Fig. 6 Anatomic model of lumbar spine used to demonstrate that the proposed method generates completions with correctly positioned vertebral landmarks.

3 Qualitative Results

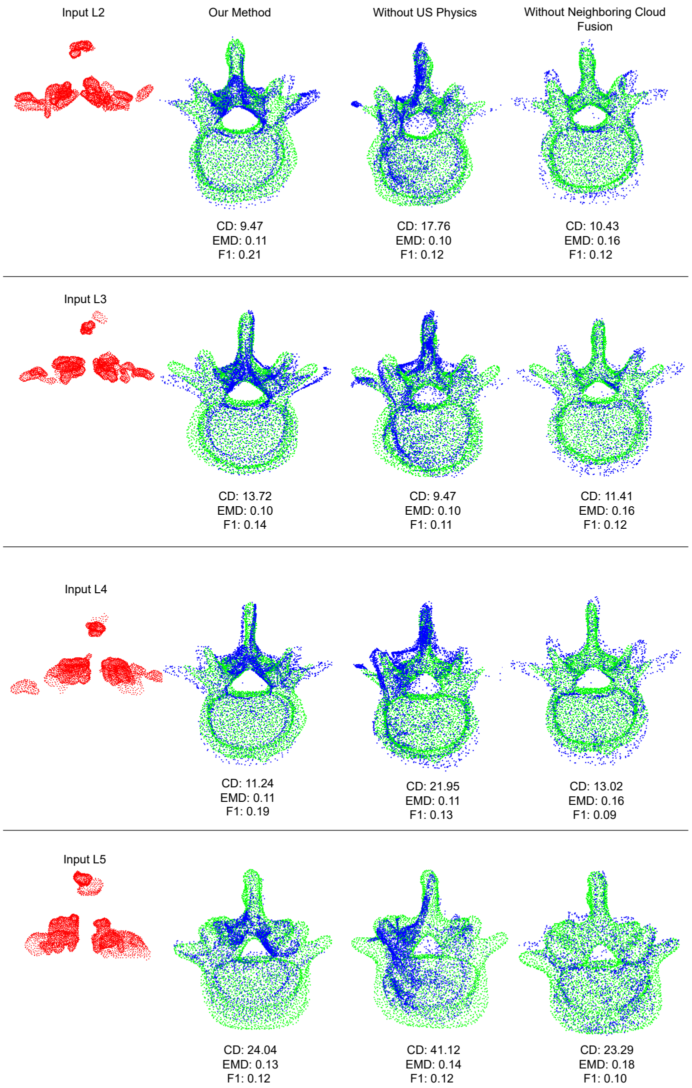

Fig. 7 Qualitative results of the proposed method as well as ablation studies on Patient 1 Ultrasound (US) data. Each row corresponds to one vertebral level. The first column displays the input to the network, while the others each show the completions in blue achieved by each network from Experiment 2 to Experiment 5 overlaid with the corresponding ground truth in green.

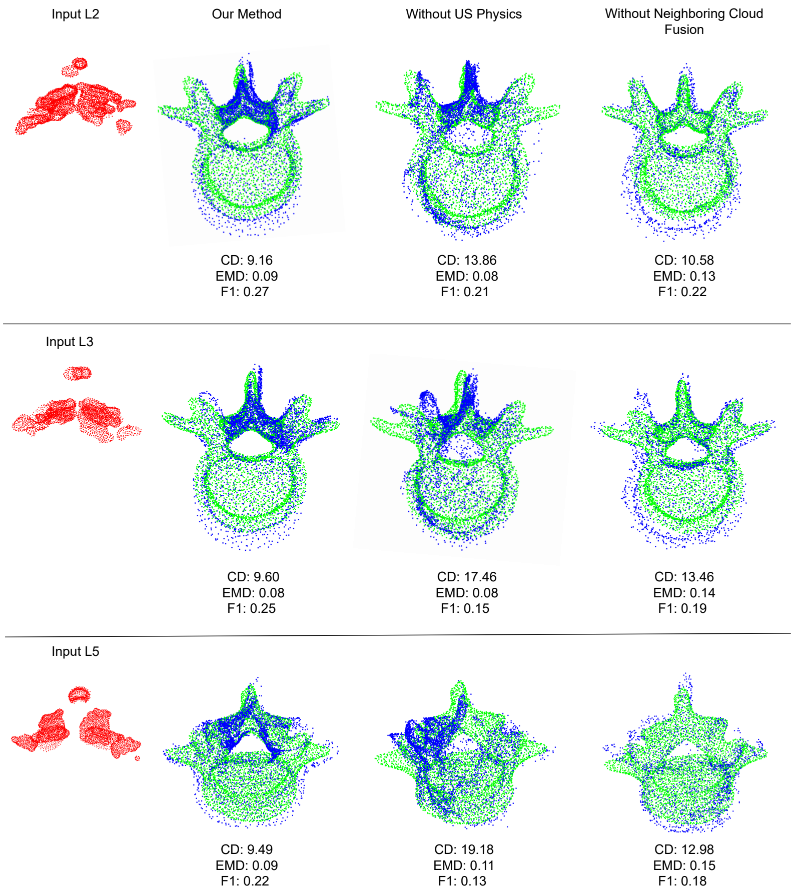

Fig. 8 Qualitative results of the proposed method as well as ablation studies on Patient 2 US data. Each row corresponds to one vertebral level. The first column displays the input to the network, while the others each show the completions in blue achieved by each network from Experiment 2 to Experiment 5 overlaid with the corresponding ground truth in green.

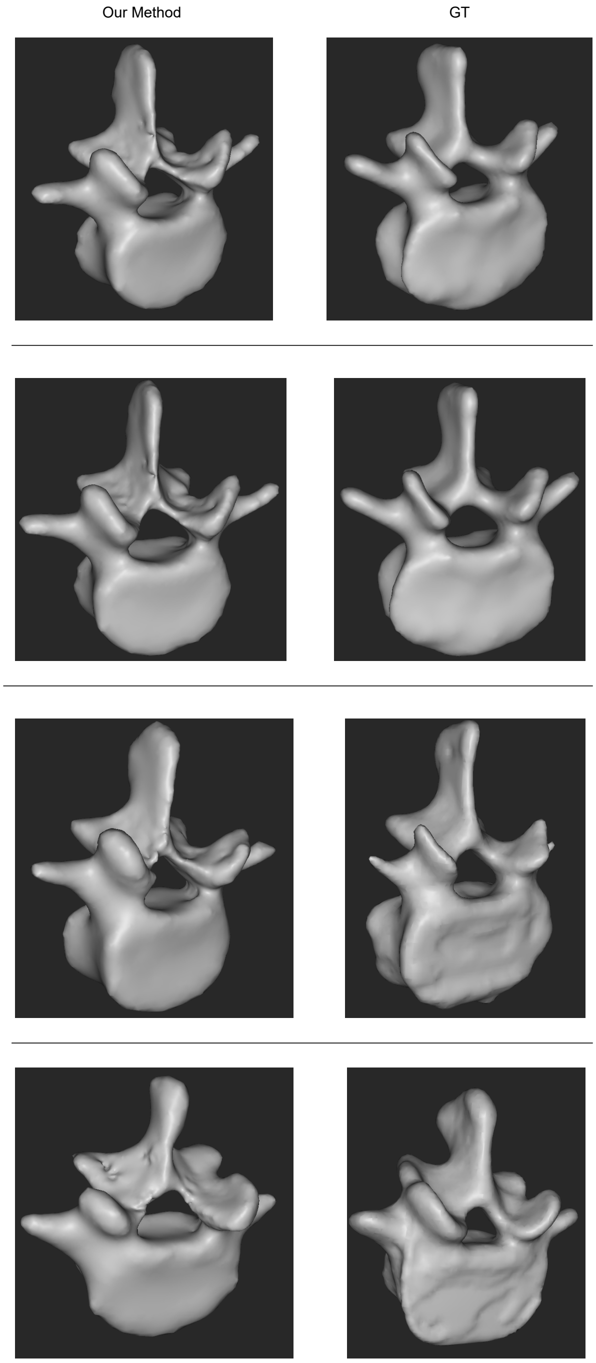

Fig. 9 Visualization of post-processed results of the proposed method on Patient 1 US data. Each row corresponds to one vertebral level, from L2 to L5. The first column displays the completed mesh while the second column shows the ground truth.

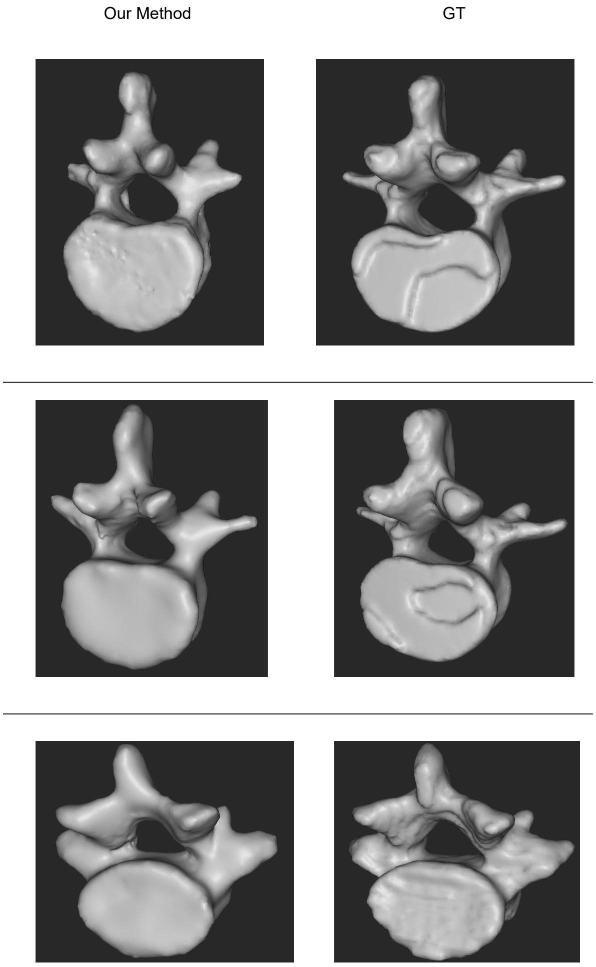

Fig. 10 Visualization of post-processed results of the proposed method on Patient 2 US data. Each row corresponds to one vertebral level, L2, L3 and L5. The first column displays the completed mesh while the second column shows the ground truth.

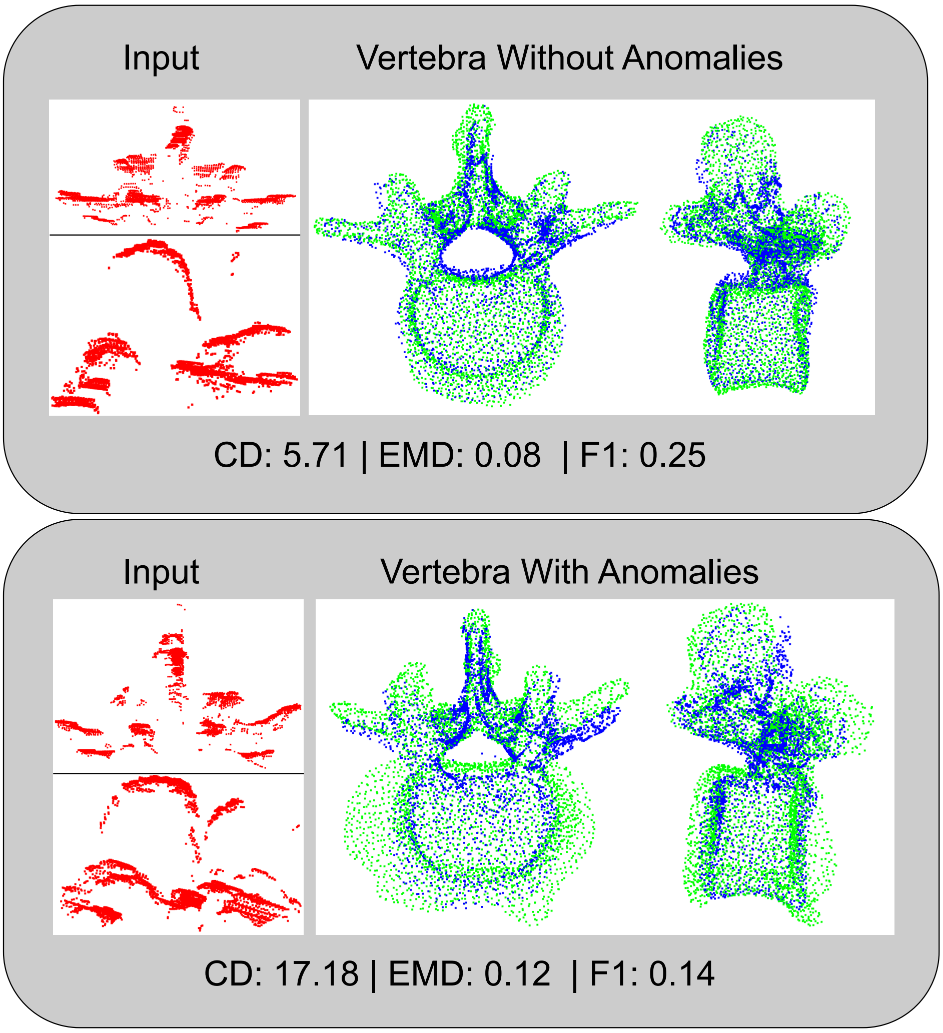

Fig. 11 Shape Completion in the example of a vertebra with and without anomaly in the vertebral body. We notice that the anomaly occurs at the level of the vertebral body. Since no indication of this anomaly is present in the input to the shape completion network, the resulting completion reconstructs a normal vertebral body.

4 Data Generation Algorithm

Algorithm 1 Synthetic Data Generation

Input: Labelmap
Output: Partial spinal pointcloud as seen in US
Extract mesh
    Run Marching Cubes algorithm on 3D label map
    Apply Gaussian Smoothing to reduce artifacts
Deform mesh
    Use DeformSpine algorithm for spine deformation
        ▷ Refer to Spine Deformation Modeling algorithm
Ray-casting
    Position the virtual rendering camera over each spinous process
    Cast rays to identify visible points
    Compute the angle of incidence for each ray and the tissue plane
    Omit points with incidence angles of $\geq 90°$
Simulate scattering
    Shift the spine mesh perpendicularly to the incident ray direction. The used shift values are found in Table 1
    Perform ray-casting on both the original and shifted spine positions
    Retain unobstructed points from the shifted mesh

Algorithm 2 Spine Deformation Modeling

Input: Lumbar spine mesh
Output: Deformed spine model
procedure DEFORMSPINE
  Define a physical model of the spine
    Model bones as rigid tissues in this model
    Model intervertebral fluids with springs
    for each vertebra $\mathcal{V}_i$ in $\{L_1, L_2, L_3, L_4, L_5\}$ **do**
      Determine points on vertebral body and facets
      Compute centroid c_i
      Define inter-body and facet joint springs between the centroids
    end for
    Spring parameters:
      Inter-vertebral fluid stiffness: 500-1000 N/m
      Number of inter-vertebral springs: 400-800
      Facet joint stiffness: 8000 N/m
      Number of facet joint springs: 200-500
      Damping coefficients: 3 N/s (inter-body), 500 N/s (facet joints)
    Connect L1 and L5 to a still constraint
  Apply forces along anterior-posterior axis
  Generate deformations in multiple directions
  Apply varying forces to L1-L5 as per predefined intervals
end procedure

References

[1] Pan L, Chen X, Cai Z, et al (2021) Variational relational point completion network. In: IEEE/CVF conference on computer vision and pattern recognition

[2] Yuan W, Khot T, Held D, et al (2018) Pcn: Point completion network. In: 2018 international conference on 3D vision (3DV)
