# Supplementary material for: Shape completion in the dark: completing vertebrae morphology from 3D ultrasound
Source: Int J Comput Assist Radiol Surg. 2024 May 15;19(7):1339–47. doi: 10.1007/s11548-024-03126-x (PMC11231015; doi:10.1007/s11548-024-03126-x)
Supplement: Supplementary file 2 — (pdf 2754 KB) [file 11548_2024_3126_MOESM2_ESM.pdf]

Appendix A Implementation Details

A.1 Synthetic data generation pipeline

A.1.1 Angle of incidence-aware Raycasting

To better understand the visual impact of the data generation pipeline on the resulting vertebral point cloud, we display a comparison of the spine mesh ray-casting with and without considering the angle of incidence in Figure A1. We observe that considering the angle of incidence leads to point cloud with more shadows, reflecting the shadowing effects in US.

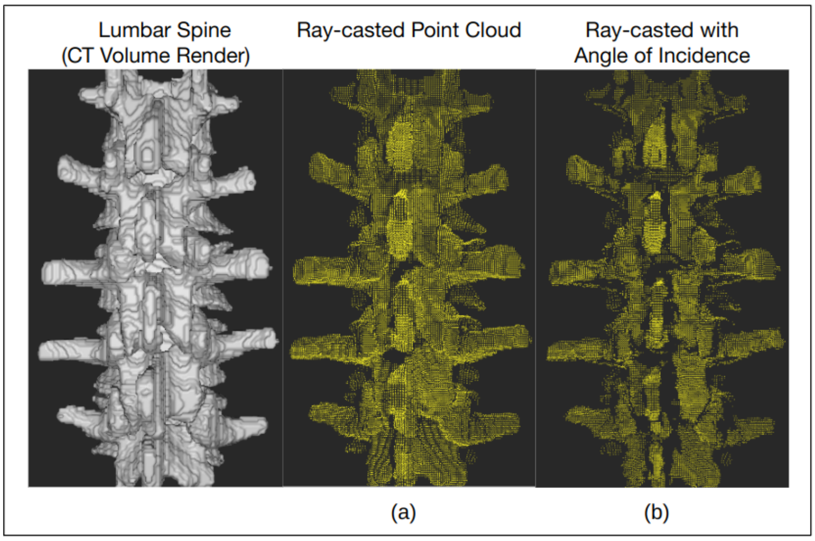

Fig. A1 Comparison of spine mesh ray-casting when (a) the angle of incidence is not considered (b) the angle of incidence is considered. The resulting point cloud contains more shadows and is, therefore, more similar to the US view of the spine.

A.1.2 Account for Ultrasound Scattering

To simulate the effect of ultrasound scattering, we have empirically defined shift values symmetrically along the lateral axis and asymmetrically on the posterior-anterior axis, detailed in Table A1. The combined mesh of the centered (blue) and shifted spine (orange) is presented on the left side of Figure A2. From this mesh, we retain points of the centered mesh unobstructed by the shift. The resultant point cloud, exhibiting more shadows, mirrors an ultrasound spine image, as visualized on Figure A2's right side. The occlusion extent and areas are shift-dependent. To diversify our synthetic output, we utilize all shift pairs from Table A1, yielding nine unique point clouds per dataset.

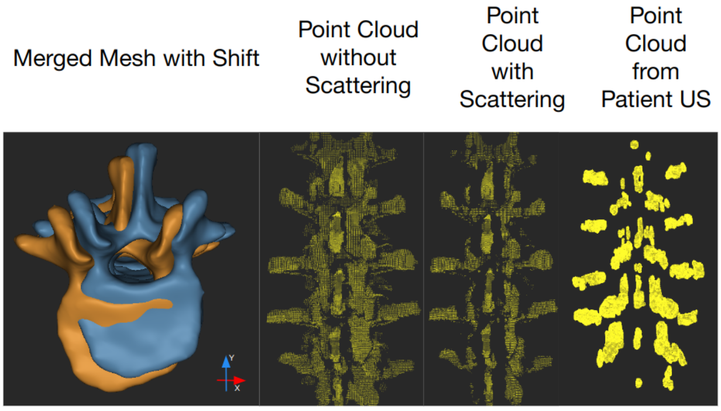

Fig. A2 Left: Example of merge between centered in blue and shifted mesh in orange by -7mm along the lateral axis (x-axis) and -5mm along the anterior-posterior axis (y-axis). Right: Comparison of ray-casted point cloud with and without scattering. We observe that the scattered version displays more shadowed areas.

	**Shift 1**	**Shift 2**	**Shift 3**
**Lateral axis (symmetrical)**	±5mm	±7mm	±10mm
**Anterior-posterior axis (asymmetrical)**	-1mm	-5mm	-10mm

Table A1 Shift values used to account for the effect of scattering in US. We use all possible pairs from these values, a total of 9 shifts per mesh for the data generation.

A.1.3 Masking spine into separate vertebrae views

To perform the neighboring cloud fusion augmentation, we sequentially place a bounding box centered on each vertebra's center of mass as visualized in Figure A3. We then extract all points within this bounding box to create the input point cloud. As exemplified in Figure A3 where the box is centered on vertebra level L3, this technique allows us to collect and merge a small number of points from neighboring vertebrae (here L2 and L4) with the points from the current vertebra.

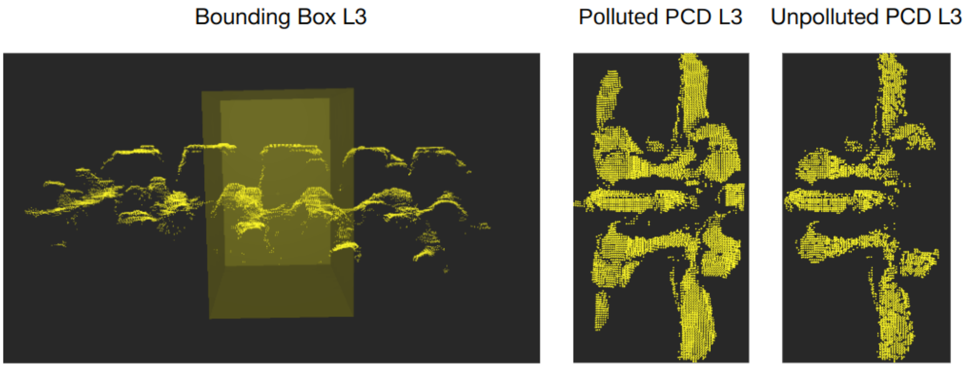

Fig. A3 The spine point cloud is masked to obtain vertebra-wise point clouds. On the left, the masking process is visualized, in which we sequentially place a bounding box centered on each vertebra and select all points within. The middle and right image compare the results of this process which we call neighboring cloud fusion with the results of omitting it.

A.2 Vertebrate Shape Completion Network Architecture

The architecture of the shape completion method consists of two networks. (1) Probabilistic modeling network (PMNet) displayed in FigureA4 and (2) Relational Enhancement Network (RENet) shown in FigureA5.

(1) The PMNet consists of two paths. The first path, known as the reconstruction path, takes a complete shape as input and reconstructs it. Simultaneously, the completion path generates a coarse point cloud from the incomplete input. These pathways are designed in an autoencoder fashion. They share weights, and the reconstruction distribution is used to regularize the completion one. (2) The RENet is designed as an encoder-decoder structure with self-attention building blocks.

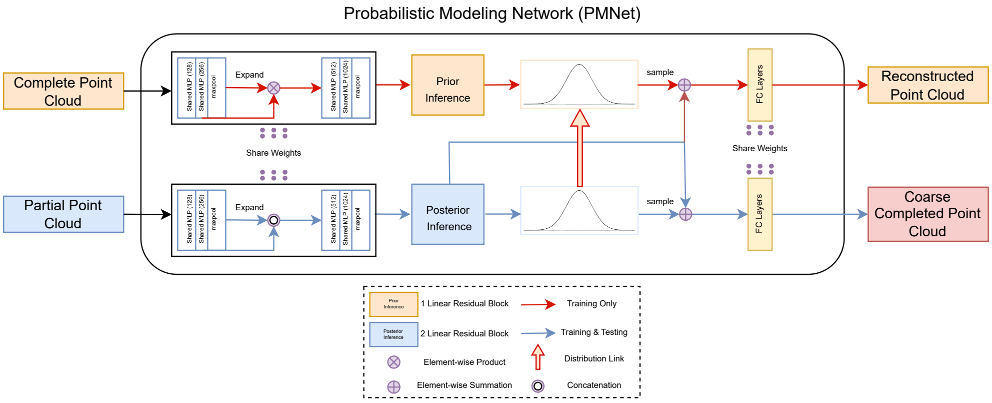

Fig. A4 Overview of VRCNet's PMNet, which generates the coarse complete point cloud at inference time based on the reconstruction latent distribution learned at training time. This figure was adapted from the original paper [7].

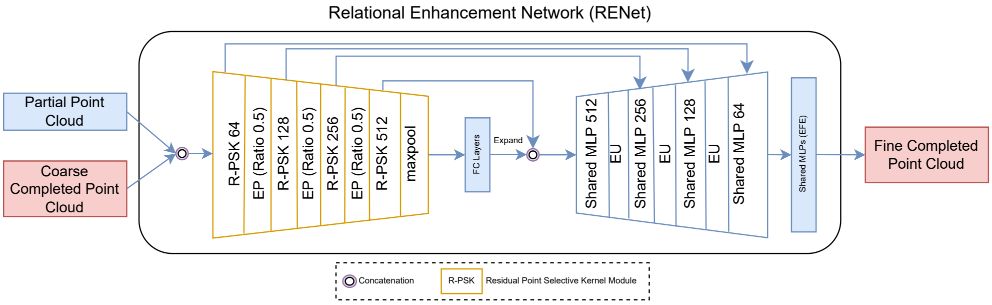

Fig. A5 Overview of VRCNet's RENet, network that generates the fine completion by using self-attention building blocks to recover local details. This figure was adapted from the original paper [7].

Appendix B Dataset details

B.1 Phantom Data

The lumbar spine phantom utilized in this work is shown in Figure B6. It contains the five lumbar vertebrae as well as the sacrum and intervertebral disks. We acquired a 3D US scan with a transverse probe orientation by using ACUSON Juniper (Siemens Healthineers, Erlangen, Germany) with a 5C1 convex probe. This probe is mounted on a 7-axis robot of the model KUKA LBR iiwa 7 R800 manipulator (KUKA Roboter GmbH, Augsburg, Germany).

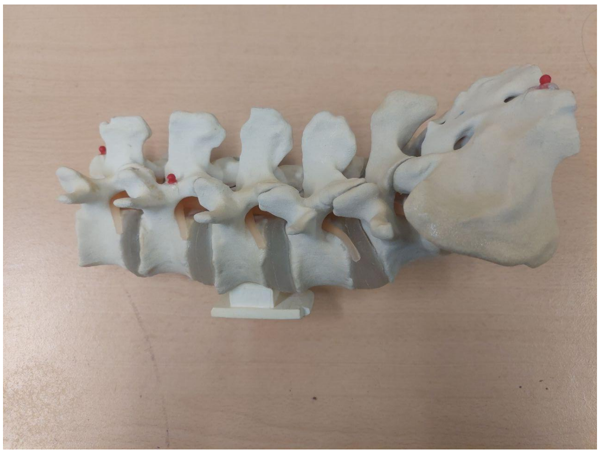

Fig. B6 Anatomic model of lumbar spine used to demonstrate that the proposed method generates completions with correctly positioned vertebral landmarks.

Appendix C Qualitative Results

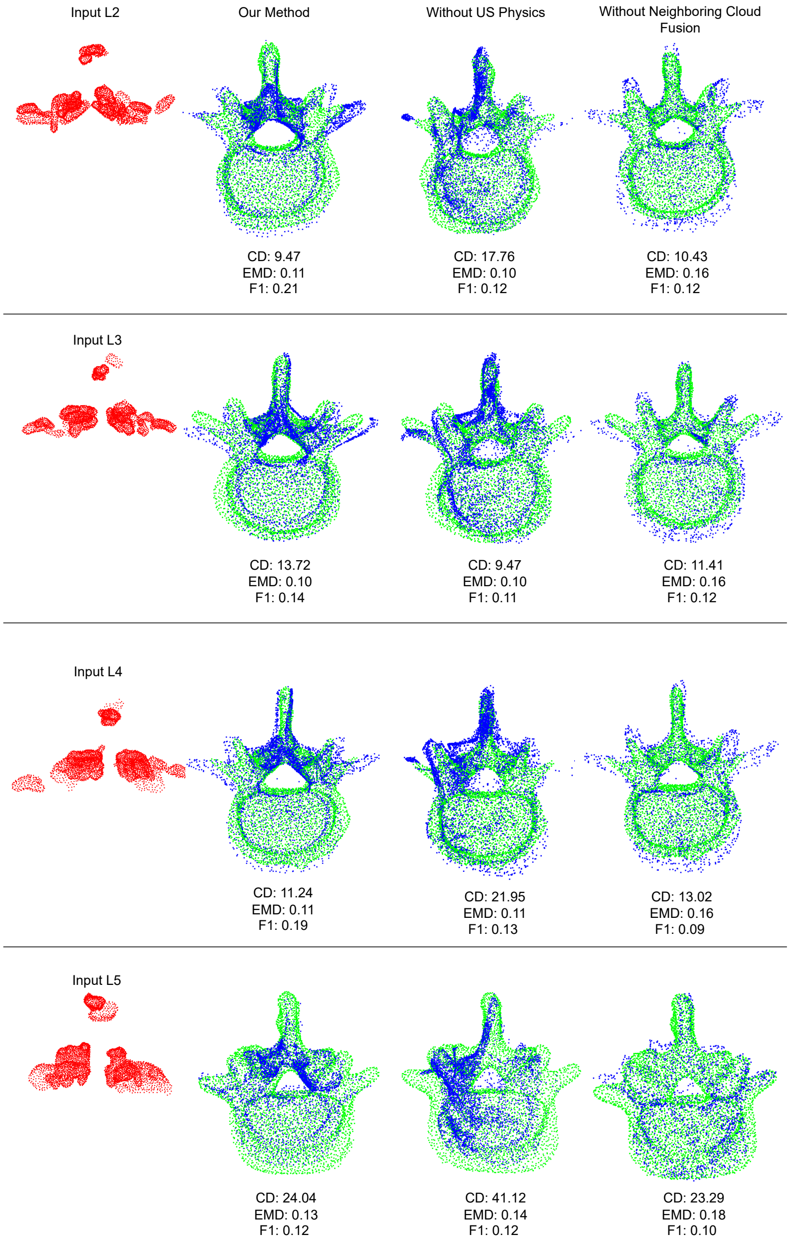

Fig. C7 Qualitative results of the proposed method as well as ablation studies on Patient 1 US data. Each row corresponds to one vertebral level. The first column displays the input to the network, while the others each show the completions in blue achieved by each network from Experiment 2 to Experiment 5 overlaid with the corresponding ground truth in green.

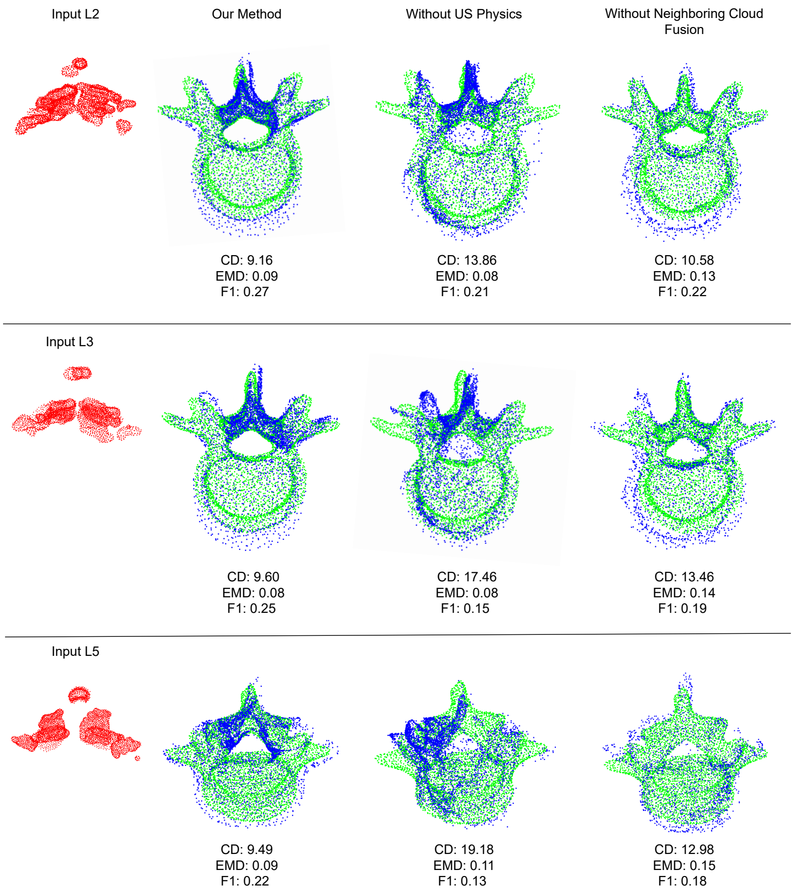

Fig. C8 Qualitative results of the proposed method as well as ablation studies on Patient 2 US data. Each row corresponds to one vertebral level. The first column displays the input to the network, while the others each show the completions in blue achieved by each network from Experiment 2 to Experiment 5 overlaid with the corresponding ground truth in green.
